# Supplementary material for: A comprehensive numerical procedure for high-intensity focused ultrasound ablation of breast tumour on an anatomically realistic breast phantom
Source: PLoS One. 2024 Oct 1;19(10):e0310899. doi: 10.1371/journal.pone.0310899 (PMC11444401; doi:10.1371/journal.pone.0310899)
Supplement: Attachment — Submitted filename: Reviewer coments.pdf [file pone.0310899.s003.pdf]

The authors have presented an appreciating simulation work of HIFU ablation of breast tumor models based on realistic anatomical structures. Unfortunately, the novelty of the work in present format is not suitable for publication. I can accept the work for publication if the authors can address the following major comments satisfactorily.

1. Please justify the novelty of the work in clinical aspect. How the proposed HIFU based therapeutic approach is beneficial over the existing non-invasive therapeutic techniques of breast cancer.
2. How the numerical work will be helpful in solving any current clinically translational research study.
3. Is the HIFU transducer parameters and other operating parameters in present problem has any clinical relevance especially the ablation period.
4. Did the authors considered the effect of large blood vessels and thermal relaxation effect of bio-tissues in present bio-heat model.
5. Realistic breast structures also includes blood vessels, and fluid glands, how this has been addressed in present model.
6. How the proposed numerical model is novel apart from the computational domain.
7. The quantity of work is not suitable for publication

Minor comments:

1. The total number of figures and tables can be reduced, Figure 1-6, 8 defines same thing i.e. the computational domain; figure 9, 10, 11 are same validation study results. Table 3 and figure 9 conveys similar information.
2. The axis label can be improved especially figure 7, 9, 15.
3. The figure formatting does not look professional, and the resolution is poor, can be improved. Figure 21 seems like a screenshot.
