# Supplementary material for: A comprehensive numerical procedure for high-intensity focused ultrasound ablation of breast tumour on an anatomically realistic breast phantom
Source: PLoS One. 2024 Oct 1;19(10):e0310899. doi: 10.1371/journal.pone.0310899 (PMC11444401; doi:10.1371/journal.pone.0310899)
Supplement: Attachment — Submitted filename: Editor_comment_revision2.pdf [file pone.0310899.s006.pdf]

This paper presents HIFU simulation study on breast 3D model with spherical lesion and fibroglandular tissue. It was emphasized that the critical need is to develop a 3D realistic breast phantom model for HIFU study. I appreciate the volume of work and analysis in this paper. However, my literature search led to another study (10.1371/journal.pone.0274801) using similar 3D model of breast by same group. In this paper, only extension is HIFU simulation of previously published model, which is a trivial step for someone working in this field. I believe modifying the applicator, repeating the same analysis and claims as in previous numerical study is not admirable scientific approach. The paper further failed to receive favorable comments from one of the reviewers. Therefore, I propose following suggestions to improve the paper:

1. Introduction:

Although some points were discussed in current introduction, but it lacks logical reasoning of extending HIFU modeling v/s MFH modeling (10.1371/journal.pone.0274801). Therefore, introduction need to add following points as well (a) what are the challenges/problems or shortcoming of previous MFH breast treatment/model?, (b) why MFH breast treatment/model is not sufficient for treatment planning and clinical workup?, (c) how a HIFU model can play a better role in solving these challenges/problems?, (d) what are the gaps/lacunae in previous implementation of HIFU models including their own model, (e) which of these gaps this study is aiming to address. Using this structure, authors could expand the scientific contribution.

2. Critical improvement or voice:

This problem or study lacks originality and critical voice. Breast lesions are not always solid mass. The real-world lesion could be non-mass enhancement and rim enhancing mass (see below figure). Therefore, a critical improvement or voice would be to compare a simulation study on HIFU treatment error/shortcomings on patient-specific modeling of non-mass enhancement, rim enhancing mass and solid mass. The study can use 3d slicer to construct realistic models of breast with (a) solid mass, (b) non-mass enhancement, and (c) rim enhancing mass, which can be shared as an 'stl' file for readers. Authors can use open-source MRI dataset to obtain sample cases of non-mass enhancement, rim enhancing mass and solid mass.

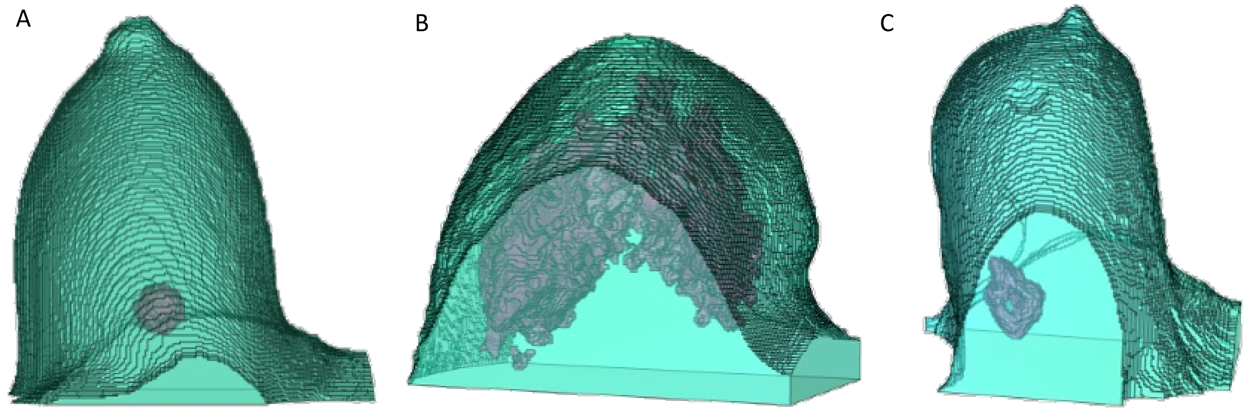

Figure: Sample images of (a) solid mass, (b) non-mass enhancement, and (c) rim enhancing mass.

Open-source database:
https://www.cancerimagingarchive.net/collection/rider-breast-mri/
https://www.cancerimagingarchive.net/collection/acrin-contralateral-breast-mr/
https://www.cancerimagingarchive.net/collection/qin-breast-dce-mri/
