# Supplementary material for: A comprehensive numerical procedure for high-intensity focused ultrasound ablation of breast tumour on an anatomically realistic breast phantom
Source: PLoS One. 2024 Oct 1;19(10):e0310899. doi: 10.1371/journal.pone.0310899 (PMC11444401; doi:10.1371/journal.pone.0310899)
Supplement: Attachment — Submitted filename: Response to reviewer2-1.docx [file pone.0310899.s004.docx]

**Response to Reviewer 2 Comments**

**Manuscript ID: PONE-D-24-02187**

**Title:** A Comprehensive Numerical Procedure for High-Intensity Focused Ultrasound Ablation of Breast Tumour on an Anatomically Realistic Breast Phantom

**Authors:** Reza Rahpeima, Chao-An Lin

**Journal:** Plos One

We thank the reviewer for these constructive comments and have made the suggested modifications to the manuscript accordingly. Based on the reviewer’s comments, new materials are added to the paper. Below is an itemized list of all the implemented changes:

**Reviewer comments:**

The authors have presented an appreciating simulation work of HIFU ablation of breast tumor models based on realistic anatomical structures. Unfortunately, the novelty of the work in present format is not suitable for publication. I can accept the work for publication if the authors can address the following major comments satisfactorily.

1. Please justify the novelty of the work in clinical aspect. How the proposed HIFU based therapeutic approach is beneficial over the existing non-invasive therapeutic techniques of breast cancer.

- **Response:** To directly address the novelty of our work in the clinical aspect, our simulation platform serves as a tool for optimizing HIFU treatment procedures. In every HIFU treatment, it is crucial to administer a sufficient dosage to ablate cancerous tissues while preventing thermal damage to surrounding healthy tissues. Clinicians can utilize this tool to test various treatment scenarios, such as adjusting the power of the transducer, determining the number of lesions needed by moving the transducer (demonstrating the platform's versatility to adapt to various treatment cases), setting the treatment duration, deciding how many sessions are needed, and other parameters. This tool aids clinicians in providing patient-specific HIFU treatments tailored to each case, considering factors like tumor size and location. Beyond its direct clinical usage, this study also lays a fundamental and mathematical foundation that could inform further development of HIFU apparatus and more.

The main purpose of the study is elaborated upon and added to the end of the introduction section as follows:

Our research addresses a significant gap in the existing literature concerning the simulation of HIFU for breast tumor ablation. While previous studies have primarily relied on 2D simulations, these models inadequately represent the three-dimensional complexities of breast anatomy and the interaction dynamics of acoustic waves within such a structure. Recognizing this limitation, our study introduces a novel all-in-one 3D FEM-based simulation approach using an anatomically realistic breast phantom. This method not only promises greater accuracy in simulating the physical phenomena involved but also provides a more reliable predictive model for clinical outcomes. By integrating detailed anatomical data into our simulations, we aim to refine the precision of HIFU therapy, optimizing its efficacy and safety in clinical applications. The introduction of a 3D model represents a substantial advancement in the simulation of therapeutic ultrasound, potentially leading to significant improvements in the treatment of breast tumors. Also, the 3D FEM simulation of acoustics, particularly in the context of HIFU, presents substantial computational demands. To address this challenge, we have developed a simulation platform that enhances computational efficiency primarily through optimized mesh generation. This optimization is crucial for making the platform viable for clinical applications, enabling the effective prediction of outcomes for various HIFU treatment strategies and required dosages. Our approach aims to balance accuracy with computational expediency, thereby facilitating more practical and rapid clinical decision-making.

Furthermore, elaborations on how a realistic simulation platform can be utilized for the optimization of treatment procedures are added at the beginning of the "Materials and Methods" section as follows:

To optimize hyperthermia treatment for breast cancer, it is essential to incorporate a patient-specific approach that begins with detailed modeling extracted from medical imaging (MRI/CT). These models help estimate the specific absorption rate (SAR) and understand the distribution of thermal energy necessary to achieve efficient tumor ablation while preserving adjacent healthy tissues. These concepts, including the illustration of different breast densities (from extremely dense to predominantly fatty), tumor depth and size, and the calculated SAR distribution, can be seen in the work by Singh et al. [23]. Such studies aids in comprehending how power is distributed and modulated to minimize the risk to surrounding healthy tissue—addressing the fringe heating phenomenon. A realistic simulation platform can be used for optimization of treatment procedure. The treatment can be tailored to manage the heat concentration precisely at the tumor location, thus sparing healthy tissues from unnecessary exposure. These realistic simulation studies not only enhance the efficacy of the treatment but also significantly reduce the collateral damage to the non-targeted areas, showcasing the pivotal role of computational modeling in personalizing cancer therapy. By extracting a patient-specific breast model from medical images, the purpose of this research is to achieve such simulation platforms mentioned that can be beneficial for pre-clinical purposes.

A comprehensive discussion section is included in the manuscript to provide further explanations. Drawbacks of our work are mentioned to highlight potential areas for future improvement. Additionally, the conclusion section is rewritten to emphasize and clarify the novelty of this work and its various applications. These revisions are highlighted in the manuscript.

We proposed several questions with their answers to further elaborate the objectives of our research as follows:

**What is already known:**

Existing studies have utilized 2D FEM simulations for HIFU, which are beneficial for initial approximations of the acoustic field and thermal effects. However, they fall short in capturing the complex interactions between the acoustic waves and the anatomical features of breast tissue, which are inherently three-dimensional.

**What is unknown:**

Prior to our study, there was a significant gap in using 3D simulations with anatomically realistic breast phantoms (ARBPs). The impact of realistic three-dimensional geometry on the efficacy and accuracy of HIFU tumor ablation has not been extensively explored.

**Why the study is important:**

Our study is critical as it provides a more realistic simulation environment which is expected to lead to better predictive models for clinical outcomes. This is particularly important for non-invasive treatments like HIFU, where the precision of tumor targeting and the avoidance of healthy tissue are paramount. Also, the 3D FEM simulation of acoustics, particularly in the context of HIFU, presents substantial computational demands. To address this challenge, we have developed a simulation platform that enhances computational efficiency primarily through optimized mesh generation. This optimization is crucial for making the platform viable for clinical applications, enabling the effective prediction of outcomes for various HIFU treatment strategies and required dosages. Our approach aims to balance accuracy with computational expediency, thereby facilitating more practical and rapid clinical decision-making.

**How existing approaches have limitations that can potentially be addressed with the current study:**

The inherent limitation of 2D models lies in their inability to capture the spatial complexities and interactions essential for accurate HIFU therapy planning, potentially leading to suboptimal therapeutic outcomes. In contrast, our 3D FEM-based model integrates a detailed anatomical representation, simulating physical phenomena with greater fidelity. This enhancement not only promises more accurate and clinically relevant insights but also maintains computational efficiency. Our simulation platform is designed to deliver these precise results without compromising on speed, making it highly suitable for the demanding context of numerical simulation in clinical settings.

**Was this approach previously tested?**

While there are instances of 3D simulations in other areas, their application to HIFU breast cancer ablation with ARBP has not been explored due to the computational complexity and the detailed anatomical data required. Our approach is novel in its application to this specific clinical scenario.

These explanations are all added to the manuscript.

2. How the present numerical work will be helpful in solving any current clinically translational research study.

- **Response:** The present numerical work provides substantial advancements in clinically translational research in the domain of HIFU for breast cancer. This study leverages a sophisticated three-dimensional Finite Element Method (3D FEM) simulation platform that markedly enhances the precision and efficacy of HIFU treatments. Here are several ways this numerical work aids in addressing current challenges in clinically translational research:

**Improved Treatment Planning:** The use of an anatomically realistic breast phantom (ARBP) and 3D simulations allows for more accurate modeling of the complex interactions between ultrasonic waves and breast tissue. This level of detail enables clinicians to plan treatments with greater precision, optimizing the focus of ultrasound waves to target tumors effectively while sparing healthy tissue. This precision is particularly crucial in treating heterogeneous structures like breast tissue, where the accurate localization of energy can significantly impact treatment outcomes.

**Customization of Therapy:** The simulation platform can be used to model various clinical scenarios, allowing researchers and clinicians to explore different treatment parameters such as the intensity of ultrasound waves, the number and position of lesions, and the duration of exposure. This ability to simulate a wide range of conditions enables the customization of therapy to individual patient anatomy and tumor characteristics, which is a core goal of translational research.

**Safety and Efficacy Evaluation:** Before clinical trials, the safety and efficacy of new treatment protocols must be rigorously evaluated. The present work allows for extensive pre-clinical testing of HIFU settings and strategies in a controlled, virtual environment, reducing the risks and uncertainties associated with direct clinical trials. By predicting outcomes and refining approaches beforehand, the study contributes to safer, more effective patient care.

**Accelerating Clinical Adoption:** By demonstrating the capabilities of 3D FEM simulations in enhancing the accuracy and predictability of HIFU treatments, this research supports the broader adoption of advanced simulation tools in clinical settings. The findings can help in regulatory approvals by providing robust data on the potential benefits and limitations of new therapeutic approaches, facilitating faster and more informed decision-making processes.

**Foundation for Future Innovations:** Beyond immediate clinical applications, the numerical work serves as a fundamental research tool that can drive further innovations in medical technology. The insights gained from detailed simulations can inform the development of next-generation HIFU devices, improved diagnostic procedures, and integrated treatment systems, expanding the scope of translational research.

3. Is the HIFU transducer parameters and other operating parameters in present problem has any clinical relevance especially the ablation period.

- **Response:** The parameters selected for the HIFU transducer and other operating parameters, including the ablation period, in our study hold significant clinical relevance, as they closely mirror the conditions encountered during actual HIFU procedures used in medical treatments. Here’s a detailed breakdown of how these parameters are pertinent to clinical practice:

**HIFU Transducer Parameters:** The design and settings of the HIFU transducer, such as frequency, shape, and focal length, directly influence the depth and precision of energy delivery into the tissue. We selected parameters that are typical of those used in clinical settings to ensure that the simulations reflect realistic therapeutic conditions. For instance, the frequency chosen affects the penetration depth and absorption characteristics of the ultrasound waves, which are critical for effectively targeting tumors at specific depths within the breast tissue.

**Ablation Period:** The duration of the ablation, or the ablation period, is particularly important for achieving the desired therapeutic effect without causing excessive damage to surrounding healthy tissues. Clinically, the ablation period must be precisely controlled to ensure that sufficient energy is delivered to induce necrosis in malignant cells while minimizing the thermal spread to adjacent tissues. Our study uses an ablation period that aligns with those commonly applied in clinical protocols, providing insights into the optimal duration needed to maximize efficacy and safety.

**Clinical Relevance of Ablation Parameters:** The parameters such as power output of the transducer and the number of sonications are adjusted based on clinical feedback and outcomes from prior treatments. These parameters are vital for clinicians to understand the therapeutic window of HIFU treatment, which balances effective tumor ablation with minimal adverse effects.

**Simulation and Validation:** By simulating the HIFU treatment under clinically relevant parameters, our study not only validates the effectiveness of the chosen settings but also allows for the exploration of parameter variations in a controlled environment. This is invaluable for clinical research, as it provides a platform for optimizing treatment protocols before they are tested in more costly and risk-associated clinical trials. Actually, the main purpose of the “3.4 Realistic clinical approach” is to show the capability of our simulation tool in real clinical approaches.

It should be emphasized that the main purpose of this research was to demonstrate the capability and versatility of our proposed numerical tool. However, in this study, we have strived to select all parameters based on realistic apparatuses that may currently be utilized in clinical settings.

4. Did the authors considered the effect of large blood vessels and thermal relaxation effect of bio-tissues in present bio-heat model.

- **Response:** Thank you for your meticulous observation. The bioheat model currently employed in our study does not explicitly incorporate the effects of large blood vessels, which significantly impact thermal dynamics due to their role in heat convection away from the heated area. In every computationally simulated model, certain aspects of the real organ cannot be precisely modeled due to the added complexity they introduce. Although our model does not explicitly include veins, their presence is indirectly considered by varying blood perfusion rates across different tissue types. These adjustments reflect areas with dense and sparse venous networks. Furthermore, our model assumes homogeneous blood perfusion, using a constant blood perfusion rate throughout the tissue. A more realistic approach, however, would involve considering a spatial distribution of blood perfusion rates. We have added a discussion of this limitation to our manuscript to ensure a comprehensive understanding of the model's constraints and implications:

In the end, some of the drawbacks of our study is mentioned here for future possible improvements. It is worth noting that the heterogeneity of blood perfusion in the breast plays a critical role in determining the temperature distribution in different parts of the tissue and also in evaluating the subsequent degree of tissue injury. In our simulation platform, the traditional Pennes Bioheat Equation, which is based on the assumption of homogeneous blood perfusion, is considered. As shown in Eq. 3, a constant value of blood perfusion rate (ω_b) is assumed for each tissue type. However, a more realistic assumption would be to consider a spatial distribution of the blood perfusion rate ω_b(x,y,z), which takes into account the heterogeneity and anisotropy of blood perfusion. Such a modified form of the Pennes Bioheat Equation has been proposed by Singh [49]. This issue can be mentioned as one of the drawbacks of our simulation platform. However, since determining such a function for the blood perfusion rate is challenging and requires experimental measurements, which are not available for human breast tissue at the moment, we are forced to consider only different constant values of blood perfusion rates corresponding to the relevant tissue type.

Also, regarding the thermal relaxation effect of bio-tissues, our study does not explicitly incorporate the effect of this. But in our paper, while the traditional use of the Arrhenius equation does not explicitly account for thermal relaxation effects, we have approached this limitation through the integration of a sophisticated bioheat transfer model and detailed temperature-time profiling. Here’s a summary of how we considered the thermal relaxation effects:

**Advanced Bioheat Transfer Model:** We utilized an advanced form of the Pennes Bioheat Equation that incorporates additional parameters to better simulate the complex heat transfer dynamics in biological tissues. This includes terms for blood perfusion and thermal conductivity that are critical in capturing how quickly tissues can dissipate heat, indirectly reflecting their thermal relaxation properties.

**Temperature-Time Profiling:** We generated detailed temperature-time profiles using our bioheat model simulations. These profiles represent the temperature dynamics within the tissue during and after the application of HIFU. By incorporating the rise and fall of tissue temperatures over time into our simulations, we implicitly included the effects of thermal relaxation. This approach allowed us to model how quickly the tissue cools down after the cessation of heat application, which is critical for calculating the real-time thermal dose received by the tissue.

**Integration with the Arrhenius Equation:** The temperature-time data obtained from our bioheat simulations were then used as input for the Arrhenius equation to calculate thermal damage. This method allowed us to assess the cumulative effect of temperature over time, including the effects of thermal relaxation, on tissue necrosis and damage.

**Validation Against Experimental Data:** We validated our simulation results against experimental data that include measurements of tissue temperature and damage under similar conditions. This validation ensured that our model accurately reflects the thermal and damage dynamics observed in actual biological tissues, thereby affirmatively incorporating the thermal relaxation effects.

5. Realistic breast structures also includes blood vessels, and fluid glands, how this has been addressed in present model.

- **Response:** Thank you for your insightful comment. In realistic breast models, incorporating blood vessels is indeed possible. However, including these elements in bioheat studies introduces complexities such as the need for fluid dynamics physics (solving Navier-Stokes equations), which would only marginally improve the model's accuracy. The primary influence of blood vessels, acting as a heat sink, is typically addressed by incorporating a blood perfusion rate parameter that represents this effect in the model. Additionally, the presence of numerous blood vessels alters the material composition of the tissue. This variation is considered by defining different tissue types, each with specific densities, thermal conductivities, and heat capacities.

The presence of fluid glands similarly impacts the thermal properties of tissue, which is why our model includes three distinct tissue types, one of which is glandular tissue. This type has thermal properties that differ from the others, detailed in the corresponding table. These methods allow us to implicitly include the effects of glands in our model.

These strategic choices, combined with our mesh optimization study, ensure that our simulation platform maintains computational efficiency while preserving its accuracy.

6. How the proposed numerical model is novel apart from the computational domain.

- **Response:** Thank you for your comment. The novelty of this work extends beyond the incorporation of an anatomically realistic breast phantom. In this study, we have undertaken a 3D simulation of HIFU and wave propagation, capturing every pertinent detail to create a highly realistic model. Additionally, the advantages of using 3D models over 2D models are thoroughly elaborated in the manuscript.

Simulating acoustics in 3D domains is computationally intensive due to the high frequency of acoustic waves and the need for very fine meshes. Typically, such models require supercomputers due to their computational demands and extensive processing time. However, our model achieves a balance between computational efficiency and precision, making it feasible on conventional computers. This is particularly significant for clinical applications, where time efficiency is crucial for patient-specific simulations. The purpose of the mesh optimization section was primarily this.

The novelty of our approach lies in several key improvements: accuracy is enhanced by incorporating precise geometric representations and adhering strictly to the governing equations; versatility is achieved through the use of 3D simulations, allowing for the modeling of complex, realistic geometries that were previously unattainable with 2D simulations; and computational efficiency is optimized to make the simulations both affordable and practical for clinical use. Furthermore, our platform's adaptability for patient-specific simulations adds significant clinical value, facilitating personalized treatment planning without sacrificing computational speed or accuracy.

7. The quantity of work is not suitable for publication.

- **Response:** Thank you for your comment. We respectfully disagree with the assessment regarding the quantity of work. This paper introduces a unique and comprehensive HIFU simulation platform that is not only technically robust but also practically relevant for clinical applications. The substantial length of the paper, 800 lines, is justified by the necessity to bridge complex mathematical formulations with real-world clinical applications effectively.

We have provided detailed explanations of the physics and governing equations to ensure the scientific rigor of our model. Additionally, we conducted thorough mesh optimization to demonstrate the robustness of our numerical simulation process. A comprehensive validation section compares our results with both previous 2D simulations and actual experimental data, underscoring the accuracy and reliability of our platform.

The simulation involves multiple intricate steps and presents a realistic approach that has direct clinical applicability. Each section of the paper—covering the detailed physics, mesh optimization, and extensive validation—is essential to articulate the connection between theoretical mathematics and practical clinical applications.

Considering both the depth and breadth of content, we believe that the quantity and significance of our work fully meet the standards expected of a scientific publication. Additionally, we have further enhanced the manuscript by adding several explanations to the 'Introduction' and 'Materials and Methods' sections. A new 'Discussion' section has also been added, which outlines the limitations of our work and suggests potential improvements for future research. Furthermore, the 'Conclusion' section has been rewritten to provide more precise inferences. These modifications are highlighted in the manuscript to facilitate easy identification.

Minor comments:

1. The total number of figures and tables can be reduced, Figure 1-6, 8 defines same thing i.e. the computational domain; figure 9, 10, 11 are same validation study results. Table 3 and figure 9 conveys similar information.

- **Response:** Thank you for your comment. As you suggested, we have reduced the number of figures. Figure 1, which presents a schematic view of the HIFU process and is not related to our computational domain, has been kept separate. Similarly, Figure 8, which details mesh density and optimized mesh size, has also been retained separately. However, following your advice, Figures 2 to 6 have been combined to create a new Figure 2 titled 'Computational Domain Details.' Additionally, Figures 9 to 11 have been merged into a new Figure 5, 'Validation Study Results.'

Regarding Table 3 and Figure 9, we believe they convey different information. As you are aware, the validation of our work with Montienthong & Rattanadecho’s study was conducted in two stages: firstly, a comparison of acoustical results, now shown in the new Figure 5 (a) and (b), and secondly, thermal results, presented in new Figure 5 (c). While Table 3 does present information from all these figures, it also provides a quantitative comparison of our results with Montienthong & Rattanadecho’s study across all stages and mentions the relevant error percentages. We believe retaining both the table and figures will greatly enhance the understanding of these errors. Consequently, the total number of figures has been reduced from 22 to 16.

2. The axis label can be improved especially figure 7, 9, 15.

- **Response:** Thank you for your comment. We believe there may have been an issue with the quality of the images as provided to you by the journal. The quality of our original figures is relatively high, and the labels are quite clear. Nonetheless, we have improved the labels and the overall quality of the figures in response to your feedback.

3. The figure formatting does not look professional, and the resolution is poor, can be improved. Figure 21 seems like a screenshot.

- **Response:** Thank you for your comment. We believe there may be an issue with the quality of the images as provided to you by the journal. The quality of our original figures is relatively high. Nevertheless, we have improved the overall quality of the figures in response to your feedback. For the figure 21 specifically, we tried to present it a little better and improved the resolution as well.
