# Supplementary material for: A comprehensive numerical procedure for high-intensity focused ultrasound ablation of breast tumour on an anatomically realistic breast phantom
Source: PLoS One. 2024 Oct 1;19(10):e0310899. doi: 10.1371/journal.pone.0310899 (PMC11444401; doi:10.1371/journal.pone.0310899)
Supplement: Attachment — Submitted filename: PLOSONE_reviewer2.docx [file pone.0310899.s005.docx]

The authors provided a detailed explanation regarding novelty of their work and also revised the manuscript accordingly. It would be satisfactory if the authors can add standard references in support of their explanations provided to justify the novelty of their work. Standard references discussing about limitations of HIFU therapy of breast tumor and clinical challenges which are addressed by present simulation study is missing in the revised manuscript. I would be happy with justification for the novelty of the work if they could provide standard references for every justification. Also simulation studies on HIFU therapy considering 3D realistic anatomical breast phantom are there in literature, below link is an example:

https://jtultrasound.biomedcentral.com/articles/10.1186/s40349-018-0111-9

Carefully site all possible references in support of explanation on how present numerical work will be helpful in solving any current clinically translational research study especially for the points: Improved Treatment Planning, Customization of Therapy, Safety and Efficacy Evaluation, Accelerating Clinical Adoption, Foundation for Future Innovations.

Carefully site all possible references in support of the explanation of clinical relevance of present HIFU transducer and other operating parameters such as ablation period, and ablation parameters.

Accordingly include all standard references in support of your study gap in the revised manuscript, so that the research question addressed in present work and its possible impact to the community is clearly visible.

The drawbacks of present model are more clearly explained in the revised manuscript. However more revision in respective concern is needed especially with the fact that consideration of inhomogeneous blood perfusion rate in bioheat model cannot ignore the effect of large blood vessels.. It is crucial to consider the effect of large blood vessels in bioheat model especially while dealing with realistic anatomical structures.

I am sorry to disagree with the explanation given in the rebuttal regarding consideration of thermal relaxation time in present bioheat model as blood perfusion and thermal conductivity. Literature shows much effort explaining the significance of considering the thermal relaxation time (τ) in Dual phase lag bioheat model especially for the case of HIFU therapy of breast tumor where intense heat concentrations are involved. Authors should consider all possible literature while explaining the limitation of their work.

I also disagree with the explanation on marginal improvement in bioheat model accuracy considering large blood vessels. Literature shows much effort to explain the significance of considering large blood vessels in bioheat model. Blood perfusion rate alone is not sufficient to consider the heat sink effect in bioheat model as in Pennes equation. Authors should clearly justify these facts in their study with appropriate references.

It is not well satisfactory that the novelty of this work extends beyond the incorporation of an anatomically realistic breast phantom in the form of a 3D simulation of HIFU and wave propagation. As there are many literature where HIFU therapy is considered on 3D model and also with realistic breast anatomy. Also proper explanation is needed on how the authors addressed the need of highly dense mesh for a 3D acoustic wave simulation in their FEM model using COMSOL with limited computational resources. Detailed explanation is needed on mesh optimization step, what size of 3D domain is considered where λ/8 mesh size is maintained, justify if the respective domain size is sufficient for a realistic anatomical breast model.

Authors can put more effort in reducing number of figure numbers for a standard publication, Acoustic pressure field and intensity defines similar thing. If the authors want to keep both acoustic intensity and pressure results in the figure then give proper justification. Also what is the need of considering the line graph and 2D map in the figures for acoustic intensity and pressure results justify.

Incorporate line numbers of the revised manuscript to address all above comments within the rebuttal.
