# Supplementary material for: A comprehensive numerical procedure for high-intensity focused ultrasound ablation of breast tumour on an anatomically realistic breast phantom
Source: PLoS One. 2024 Oct 1;19(10):e0310899. doi: 10.1371/journal.pone.0310899 (PMC11444401; doi:10.1371/journal.pone.0310899)
Supplement: Attachment — Submitted filename: Response to reviewer2.docx [file pone.0310899.s007.docx]

**Response to Reviewer 2 Comments**

**Manuscript ID: PONE-D-24-02187R1**

**Title:** A Comprehensive Numerical Procedure for High-Intensity Focused Ultrasound Ablation of Breast Tumour on an Anatomically Realistic Breast Phantom

**Authors:** Reza Rahpeima, Chao-An Lin

**Journal:** Plos One

We thank the reviewer for these constructive comments and have made the suggested modifications to the manuscript accordingly. Based on the reviewer’s comments, new materials are added to the paper. Below is an itemized list of all the implemented changes:

**Reviewer comments:**

1. The authors provided a detailed explanation regarding novelty of their work and also revised the manuscript accordingly. It would be satisfactory if the authors can add standard references in support of their explanations provided to justify the novelty of their work. Standard references discussing about limitations of HIFU therapy of breast tumor and clinical challenges which are addressed by present simulation study is missing in the revised manuscript. I would be happy with justification for the novelty of the work if they could provide standard references for every justification. Also, simulation studies on HIFU therapy considering 3D realistic anatomical breast phantom are there in literature, below link is an example:

<https://jtultrasound.biomedcentral.com/articles/10.1186/s40349-018-0111-9>

- **Response:** We modified the introduction by adding complementary information about the limitations of HIFU therapy for breast tumors and the clinical challenges in previous studies, as well as how our study addresses these limitations as the novelty of our study. Each of the mentioned challenges is cited by the relevant references as you suggested (six more references were added to this part). Also, your mentioned reference is added to the introduction part and further explanations are added. The implementations are as follows:

In 2021, Singh et al. conducted a numerical study to investigate the effectiveness of pulsed ultrasound in assisting thermo-therapy for the ablation of subsurface tumors, focusing on the temperature variations and thermal dose distribution achieved during the treatment [20]. Okita et al. [20] investigated the effects of breast structure on high-intensity focused ultrasound (HIFU) focal errors. They conducted numerical simulations of breast cancer HIFU ablation using digital breast phantoms constructed from MRI data of 12 patients. However, this study does not specifically show temperature distribution and the resulting amount of necrotized tissue. Instead, it emphasizes how different breast tissue structures can cause focal errors in HIFU treatments, highlighting the need for precise targeting and adjustment based on individual anatomical differences.

Our research addresses a significant gap in the existing literature concerning the simulation of HIFU for breast tumor ablation. While previous studies have primarily relied on 2D simulations, these models inadequately represent the three-dimensional complexities of breast anatomy and the interaction dynamics of acoustic waves within such a structure. Yadav et al. highlighted the transition from 2D to more advanced 3D models using k-wave simulation, underscoring the limitations of earlier 2D simulations in accurately representing complex breast anatomy and treatment dynamics [21]. Badawe et al. focused on the effect of pulsed HIFU on breast cancer and compared 2D and 3D simulation paradigms, pointing out the inadequacies of 2D models in capturing the three-dimensional complexities of breast tumor anatomy and treatment effects [22]. Recognizing this limitation, our study introduces a novel all-in-one 3D FEM-based simulation approach using an anatomically realistic breast phantom. This method not only promises greater accuracy in simulating the physical phenomena involved but also provides a more reliable predictive model for clinical outcomes. By integrating detailed anatomical data into our simulations, we aim to refine the precision of HIFU therapy, optimizing its efficacy and safety in clinical applications.

The development of an all-in-one simulation platform that models all physical aspects of HIFU treatment—including sound intensity, temperature achieved, and necrotized tissue—using patient-specific breast models from medical images can serve as a powerful tool for pre-clinical evaluation and prediction. This platform addresses several key limitations and challenges in HIFU therapy. Traditional methods may struggle with accurately targeting tumors due to variability in patient anatomy [23], but patient-specific simulations enhance precision by accounting for individual anatomical differences, leading to more effective and safer treatments. The effectiveness of HIFU therapy depends on delivering the correct thermal dose to the tumor while sparing surrounding tissues, and realistic models predict thermal distribution more accurately, ensuring optimal dosing and reducing the risk of damage to healthy tissues [21]. Additionally, standard treatment protocols may not be suitable for all patients due to differences in tissue properties and tumor location [24]. Patient-specific simulations allow for tailored treatment planning, optimizing parameters such as intensity, duration, and focus of the ultrasound waves to maximize therapeutic outcomes. This comprehensive approach ultimately guides clinicians in choosing the optimal treatment dose and strategies.

Also, the 3D FEM simulation of acoustics, particularly in the context of HIFU, presents substantial computational demands [25].

Line 97 to 134.

2. Carefully site all possible references in support of explanation on how present numerical work will be helpful in solving any current clinically translational research study especially for the points: Improved Treatment Planning, Customization of Therapy, Safety and Efficacy Evaluation, Accelerating Clinical Adoption, Foundation for Future Innovations.

- **Response:** We added a paragraph at the end of the discussion section, explaining how the present numerical work will be beneficial in several areas: Improved Treatment Planning, Customization of Therapy, Safety and Efficacy Evaluation, Accelerating Clinical Adoption, and Foundation for Future Innovations. Each explanation is supported by a relevant reference, as requested. The implemented changes are as follows:

In the end, we explained here how the present numerical work will be helpful in addressing current clinical translational research studies. Our 3D FEM-based numerical procedure improves HIFU treatment planning by accurately simulating acoustic fields, thermal distribution, and necrotic tissue formation in realistic breast phantoms. This helps clinicians tailor HIFU parameters to each patient's anatomy, resulting in more effective and personalized treatment plans [59]. Using patient-specific breast models from T1-weighted MRIs customizes HIFU therapy by allowing clinicians to optimize ultrasound wave parameters. This approach addresses patient anatomy and tumor variability, improving therapeutic outcomes while minimizing damage to healthy tissues [12]. The numerical platform simulates thermal effects and tissue necrosis, accurately predicting temperature and damage. This ensures safer and more effective HIFU treatments by mitigating overheating risks and collateral damage [59]. Validation against experimental data and literature confirms our model's reliability and accuracy. Boosting clinical confidence in 3D simulations for HIFU treatment planning and outcome prediction, encourages HIFU adoption in clinical practice [60]. Our comprehensive 3D FEM-based simulation approach sets a foundation for future innovations in HIFU therapy. The ability to simulate complex interactions within realistic anatomical structures opens avenues for developing advanced HIFU techniques and integrating them with other therapeutic modalities [22]. This platform can also be used for pre-clinical testing of new HIFU devices and protocols, fostering continuous improvement in the field.

Line 760 to 776

3. Carefully site all possible references in support of the explanation of clinical relevance of present HIFU transducer and other operating parameters such as ablation period, and ablation parameters.

- **Response:** We cited all of the references in support of the explanation of clinical relevance of each of the parameters and procedures used in our paper. These references are mentioned in the manuscript as follows:

the tumour is modelled as a sphere with a 10 mm diameter. This kind of tumour, which is tiny and in the early stages of cancer growth, is categorized as T1c [40].

Line 268 and 269

The amplitude of this input signal is considered to be 0.1 MPa. According to Diaz et al. [41], the solutions reported for a similar HIFU apparatus of input below $p_{0}=0.35$ MPa remains in the linear regime.

Line 278 to 280

Also, the acoustic frequency was set to 1.5 MHz, which is a standard frequency for transducers used in HIFU therapeutic applications [42].

Line 285 and 286

These exposure and therapy durations are similar to those in the Montienthong and Rattanadecho study [3] and can be further ratified by other clinical studies [43].

Line 294 and 295

The equations for these boundary conditions are provided [36]

Line 319

Thermophysical and acoustic properties of different materials [1, 8, 44, 49-55].

Line 332

The use of mesh sizes λ/4 for solving acoustic equations is mentioned in the context of high-precision finite element modeling [56].

Line 345 to 347

Guidelines from COMSOL Multiphysics emphasize using even smaller mesh sizes than λ/4 for reliable wave propagation simulations in high-intensity areas, indicating that finer meshes like λ/8 provide better accuracy [57].

Line 348 to 350

This HIFU therapy procedure of generating multiple lesions and moving the transducer focus, along with the durations considered, is based on previous clinical approaches [59, 60].

Line 560 to 562

4. Accordingly include all standard references in support of your study gap in the revised manuscript, so that the research question addressed in present work and its possible impact to the community is clearly visible.

- **Response:** The research gap and how our paper addresses each of them are mentioned in the introduction section, with multiple references added to support our statements. The implementations in the manuscript are detailed below:

Our research addresses a significant gap in the existing literature concerning the simulation of HIFU for breast tumor ablation. While previous studies have primarily relied on 2D simulations, these models inadequately represent the three-dimensional complexities of breast anatomy and the interaction dynamics of acoustic waves within such a structure. Yadav et al. highlighted the transition from 2D to more advanced 3D models using k-wave simulation, underscoring the limitations of earlier 2D simulations in accurately representing complex breast anatomy and treatment dynamics [21]. Badawe et al. focused on the effect of pulsed HIFU on breast cancer and compared 2D and 3D simulation paradigms, pointing out the inadequacies of 2D models in capturing the three-dimensional complexities of breast tumor anatomy and treatment effects [22]. Recognizing this limitation, our study introduces a novel all-in-one 3D FEM-based simulation approach using an anatomically realistic breast phantom. This method not only promises greater accuracy in simulating the physical phenomena involved but also provides a more reliable predictive model for clinical outcomes. By integrating detailed anatomical data into our simulations, we aim to refine the precision of HIFU therapy, optimizing its efficacy and safety in clinical applications.

The development of an all-in-one simulation platform that models all physical aspects of HIFU treatment—including sound intensity, temperature achieved, and necrotized tissue—using patient-specific breast models from medical images can serve as a powerful tool for pre-clinical evaluation and prediction. This platform addresses several key limitations and challenges in HIFU therapy. Traditional methods may struggle with accurately targeting tumors due to variability in patient anatomy [23], but patient-specific simulations enhance precision by accounting for individual anatomical differences, leading to more effective and safer treatments. The effectiveness of HIFU therapy depends on delivering the correct thermal dose to the tumor while sparing surrounding tissues, and realistic models predict thermal distribution more accurately, ensuring optimal dosing and reducing the risk of damage to healthy tissues [21]. Additionally, standard treatment protocols may not be suitable for all patients due to differences in tissue properties and tumor location [24]. Patient-specific simulations allow for tailored treatment planning, optimizing parameters such as intensity, duration, and focus of the ultrasound waves to maximize therapeutic outcomes. This comprehensive approach ultimately guides clinicians in choosing the optimal treatment dose and strategies.

Also, the 3D FEM simulation of acoustics, particularly in the context of HIFU, presents substantial computational demands [25]. To address this challenge, we have developed a simulation platform that enhances computational efficiency primarily through optimized mesh generation. This optimization is crucial for making the platform viable for clinical applications, enabling the effective prediction of outcomes for various HIFU treatment strategies and required dosages. Our approach aims to balance accuracy with computational expediency, thereby facilitating more practical and rapid clinical decision-making.

To obtain results as close to reality as possible, this study utilizes a physically realistic 3D model and an anatomically realistic breast phantom (ARBP). The ARBP is created from T1-weighted MRIs of patients in the prone position [26]. The use of an ARBP ensures that the simulation considers the complex anatomy of the breast, leading to more accurate and clinically relevant results. Furthermore, a more realistic HIFU ablation procedure was performed in this study, involving several lesion points at different areas of the tumour. The cumulative effect of these several lesion points on the fraction of necrotic tissue was evaluated, demonstrating the capability of this numerical platform for patient-specific simulation of real clinical HIFU therapies. Additionally, different abnormal tissue geometries are considered to investigate the effect of tumor types and shapes on HIFU ablation outcomes.

Line 106 to 148

5. The drawbacks of present model are more clearly explained in the revised manuscript. However more revision in respective concern is needed especially with the fact that consideration of inhomogeneous blood perfusion rate in bioheat model cannot ignore the effect of large blood vessels. It is crucial to consider the effect of large blood vessels in bioheat model especially while dealing with realistic anatomical structures.

**Response:** We acknowledge that the presence of large blood vessels can significantly impact heat distribution and thus the outcome of HIFU therapy. In our study, we employed the Pennes bioheat transfer equation, which traditionally incorporates a perfusion term accounting for the cooling effect of blood flow. While this model is effective for small vessels and capillaries, it does not explicitly consider the significant thermal sinks created by large blood vessels due to convective heat transfer.

To address this limitation, future work could incorporate a more detailed vascular model that includes major blood vessels within the breast tissue. This enhancement would involve segmenting large blood vessels from MRI data and integrating their thermal effects into the simulation. Doing so would provide a more accurate representation of thermal fields during HIFU treatment, which is particularly critical for realistic anatomical structures.

However, it is important to note that each simulation model has its limitations based on available resources. We aimed to develop a simulation platform that can be run on conventional computers, and incorporating such complex vessel structures and relevant fluid flow physics would result in extensive computational burden. The study you mentioned before that utilized several realistic MRI breast images (https://jtultrasound.biomedcentral.com/articles/10.1186/s40349-018-0111-9) does not incorporate vascular structures or large blood vessels as well. This explanation of limitation of our study and the potential future research directions along with the relevant references are added to the discussion section of the manuscript as follows:

Also, presence of large blood vessels can significantly impact heat distribution and the outcome of HIFU therapy due to their ability to act as thermal sinks through convective heat transfer, which is critical to consider for accurate treatment planning [65-68]. Our study used the Pennes bioheat transfer equation, which incorporates a perfusion term accounting for the cooling effect of blood flow. While this model is effective for small vessels and capillaries, it does not explicitly consider the significant thermal sinks created by large blood vessels due to convective heat transfer. To address this, future work could include a detailed vascular model by segmenting large blood vessels from MRI data and integrating their thermal effects into the simulation. This would provide a more accurate representation of thermal fields during HIFU treatment, especially for realistic anatomical structures, but would, of course, incur further computational costs.

Line 720 to 729

6. I am sorry to disagree with the explanation given in the rebuttal regarding consideration of thermal relaxation time in present bioheat model as blood perfusion and thermal conductivity. Literature shows much effort explaining the significance of considering the thermal relaxation time (τ) in Dual phase lag bioheat model especially for the case of HIFU therapy of breast tumor where intense heat concentrations are involved. Authors should consider all possible literature while explaining the limitation of their work.

- **Response:** We acknowledge the reviewer's comment regarding the importance of considering thermal relaxation time (τ) in the bioheat model. Our study does not explicitly incorporate thermal relaxation effects. The traditional use of the Arrhenius equation does not account for these effects, which is a limitation of our current work. However, we have attempted to address this through the integration of a sophisticated bioheat transfer model and detailed temperature-time profiling, as explained in our previous response to your review. Unfortunately, as you noted, this approach is not as accurate as explicitly considering thermal relaxation time. This limitation of our work has been added to the discussion section, along with relevant references. We also outline this possible area for future improvements. The added paragraph is as follows:

One important aspect of bioheat modeling is the consideration of thermal relaxation time (τ) in the Dual Phase Lag (DPL) bioheat model, especially for HIFU therapy of breast tumors where intense heat concentrations are involved. Our study does not explicitly incorporate thermal relaxation effects, and the traditional use of the Arrhenius equation does not account for these effects, which is a limitation. However, we have attempted to address this through the integration of a sophisticated bioheat transfer model and detailed temperature-time profiling. In future studies, incorporating the DPL bioheat model to explicitly consider thermal relaxation time will enhance the accuracy of simulations by accounting for the lag in heat transfer response. This is critical for HIFU therapy of breast tumors, as it better simulates the complex heat dynamics involved. Thermal relaxation time in the DPL model represents the delay in heat conduction, capturing the lag between heat application and temperature change, which is particularly important in scenarios with quick temperature changes, such as HIFU therapy. Studies by Bhowmik et al. and Paul et al. highlight the significance of considering thermal relaxation in bioheat models [69, 70].

Line 730 to 741

7. I also disagree with the explanation on marginal improvement in bioheat model accuracy considering large blood vessels. Literature shows much effort to explain the significance of considering large blood vessels in bioheat model. Blood perfusion rate alone is not sufficient to consider the heat sink effect in bioheat model as in Pennes equation. Authors should clearly justify these facts in their study with appropriate references.

**Response:** The explanations regarding the effect of large blood vessels are mentioned in the 5^th^ answer and relevant references are added as well.

8. It is not well satisfactory that the novelty of this work extends beyond the incorporation of an anatomically realistic breast phantom in the form of a 3D simulation of HIFU and wave propagation. As there are many literature where HIFU therapy is considered on 3D model and also with realistic breast anatomy.

- **Response:** The novelty of this study is elaborated in detail at the end of the introduction section. The key innovations include: considering a 3D domain instead of 2D, developing a comprehensive all-in-one simulation platform capable of considering various physical phenomena from beginning to end, creating a computationally efficient platform through mesh optimization that can be utilized by conventional computers, and utilizing an anatomically realistic breast phantom to demonstrate patient-specific applications of this platform. Additionally, the consideration of an all-in-one simulation that couples all the various physical phenomena involved in the HIFU ablation process—including pressure acoustics, heat transfer, and subsequent calculation of the amount of necrotized tissue—by using a realistic 3D breast phantom has not been extensively done in previous literature. These explanations, along with the relevant references, have been added to the manuscript as follows:

Our research addresses a significant gap in the existing literature concerning the simulation of HIFU for breast tumor ablation. While previous studies have primarily relied on 2D simulations, these models inadequately represent the three-dimensional complexities of breast anatomy and the interaction dynamics of acoustic waves within such a structure. Yadav et al. highlighted the transition from 2D to more advanced 3D models using k-wave simulation, underscoring the limitations of earlier 2D simulations in accurately representing complex breast anatomy and treatment dynamics [22]. Badawe et al. focused on the effect of pulsed HIFU on breast cancer and compared 2D and 3D simulation paradigms, pointing out the inadequacies of 2D models in capturing the three-dimensional complexities of breast tumor anatomy and treatment effects [23]. Recognizing this limitation, our study introduces a novel all-in-one 3D FEM-based simulation approach using an anatomically realistic breast phantom. This method not only promises greater accuracy in simulating the physical phenomena involved but also provides a more reliable predictive model for clinical outcomes. By integrating detailed anatomical data into our simulations, we aim to refine the precision of HIFU therapy, optimizing its efficacy and safety in clinical applications.

The development of an all-in-one simulation platform that models all physical aspects of HIFU treatment—including sound intensity, temperature achieved, and necrotized tissue—using patient-specific breast models from medical images can serve as a powerful tool for pre-clinical evaluation and prediction. This platform addresses several key limitations and challenges in HIFU therapy. Traditional methods may struggle with accurately targeting tumors due to variability in patient anatomy [24], but patient-specific simulations enhance precision by accounting for individual anatomical differences, leading to more effective and safer treatments. The effectiveness of HIFU therapy depends on delivering the correct thermal dose to the tumor while sparing surrounding tissues, and realistic models predict thermal distribution more accurately, ensuring optimal dosing and reducing the risk of damage to healthy tissues [22]. Additionally, standard treatment protocols may not be suitable for all patients due to differences in tissue properties and tumor location [25]. Patient-specific simulations allow for tailored treatment planning, optimizing parameters such as intensity, duration, and focus of the ultrasound waves to maximize therapeutic outcomes. This comprehensive approach ultimately guides clinicians in choosing the optimal treatment dose and strategies.

Also, the 3D FEM simulation of acoustics, particularly in the context of HIFU, presents substantial computational demands [26]. To address this challenge, we have developed a simulation platform that enhances computational efficiency primarily through optimized mesh generation. This optimization is crucial for making the platform viable for clinical applications, enabling the effective prediction of outcomes for various HIFU treatment strategies and required dosages. Our approach aims to balance accuracy with computational expediency, thereby facilitating more practical and rapid clinical decision-making.

To obtain results as close to reality as possible, this study utilizes a physically realistic 3D model and an anatomically realistic breast phantom (ARBP). The ARBP is created from T1-weighted MRIs of patients in the prone position [27]. The use of an ARBP ensures that the simulation considers the complex anatomy of the breast, leading to more accurate and clinically relevant results. Furthermore, a more realistic HIFU ablation procedure was performed in this study, involving several lesion points at different areas of the tumour. The cumulative effect of these several lesion points on the fraction of necrotic tissue was evaluated, demonstrating the capability of this numerical platform for patient-specific simulation of real clinical HIFU therapies. Additionally, different abnormal tissue geometries are considered to investigate the effect of tumor types and shapes on HIFU ablation outcomes.

In this paper, we present a comprehensive approach to predict the outcome of the HIFU process using an ARBP. The simulation process involves several steps, starting with the generation of an acoustic wave and the calculation of the acoustic intensity distribution in the tissue phantom. The absorbed acoustic energy is then utilized as the heat source in the bioheat transfer model, allowing for the calculation of the amount of heat produced and the resulting increase in temperature in the tumour area. Finally, the fraction of necrotic tissue can be determined based on the temperature increase.

Line 106 to 154

9. Also, proper explanation is needed on how the authors addressed the need of highly dense mesh for a 3D acoustic wave simulation in their FEM model using COMSOL with limited computational resources. Detailed explanation is needed on mesh optimization step, what size of 3D domain is considered where λ/8 mesh size is maintained, justify if the respective domain size is sufficient for a realistic anatomical breast model.

- **Response:** The explanation on how we addressed the need for a highly dense mesh for a 3D simulation of our FEM model is added to the manuscript. Further detailed explanations are also present in the manuscript. Regarding your question about where the λ/8 mesh size is maintained: this mesh size is applied to the tumor area to resolve the sharp pressure gradient at this location. An element growth rate of 1.2 is used, which gradually increases the mesh size as it moves further from the tumor, but it never exceeds the λ/4 minimum needed for accurately solving acoustic equations. These modifications are applied to the manuscript and relevant references are added to support it as well. Th emodifications are as follows:

For solving acoustic equations with high precision, a very fine mesh set is applied to the ARBP and the surrounding water with a maximum mesh size equal to λ/4 (where λ is the wavelength). The use of mesh sizes λ/4 for solving acoustic equations is mentioned in the context of high-precision finite element modeling [57]. Also, to accurately resolve the sharp pressure gradient in the focal region, the model uses an even finer mesh with a maximum mesh size of λ/8 for the tumour tissue. Guidelines from COMSOL Multiphysics emphasize using even smaller mesh sizes than λ/4 for reliable wave propagation simulations in high-intensity areas, indicating that finer meshes like λ/8 provide better accuracy [58]. So, the λ/8 mesh size is applied to the tumor area to resolve the sharp pressure gradient at this location, and an element growth rate of 1.2 is used, which gradually increases the mesh size as it moves further from the tumor. The mesh size becomes larger in regions farther from the tumor, as these regions have a more negligible effect on the calculation of correct results.

Line 344 to 354

As mentioned before, the need for a highly dense mesh in the 3D simulation of HIFU in the FEM model is addressed by using two different sets of mesh grids. One set consists of very fine meshes for the simulation of acoustic waves, optimized separately by applying the necessary mesh sizes for high-precision acoustic simulation based on available guidelines and a comprehensive study balancing computational cost and simulation accuracy. The other set uses coarser meshes for the bioheat simulation, which does not require the very fine mesh used for acoustic waves. A similar comprehensive study was conducted for this mesh set to balance computational cost and simulation accuracy as well. By incorporating this approach, we were able to achieve a high-accuracy 3D simulation model of the entire real-size breast phantom.

Line 385 to 392

10. Authors can put more effort in reducing number of figure numbers for a standard publication, Acoustic pressure field and intensity defines similar thing. If the authors want to keep both acoustic intensity and pressure results in the figure then give proper justification. Also what is the need of considering the line graph and 2D map in the figures for acoustic intensity and pressure results justify.

- **Response:**

**The Justification for Including Both Acoustic Pressure and Intensity Results**

a. **Complementary Information:** Although acoustic pressure and intensity are related, they provide complementary information about the acoustic field. Acoustic pressure represents the oscillating component of the wave, whereas acoustic intensity represents the energy flow per unit area. Both are crucial for a comprehensive understanding of the acoustic field and its effects on tissue.
b. **Verification and Validation:** Including both pressure and intensity results allows for better verification and validation of the simulation. It ensures that the model accurately captures both the amplitude and energy aspects of the acoustic field.

**Justification for Including Line Graphs and 2D Maps**

a. **2D Maps for Spatial Distribution:** The 2D maps provide a visual representation of the spatial distribution of acoustic pressure and intensity across the tissue. This is essential for identifying focal zones and understanding the overall pattern of the acoustic field.
b. **Line Graphs for Detailed Analysis:** Line graphs (1D plots) offer a detailed quantitative analysis along specific lines of interest. They allow for the precise examination of variations in pressure and intensity, which is critical for evaluating the sharp gradients and focal points that are not as easily discernible in 2D maps. Also, will show in detail that what exactly is happening in the tumor location.
c. **Enhanced Clarity and Comprehension:** Combining both visual formats enhances the clarity and comprehension of the results. The 2D maps provide an overview, while the line graphs offer detailed insights, making it easier to interpret the data and draw accurate conclusions.

**Conclusion**

In conclusion, retaining both the acoustic pressure and intensity results, along with the inclusion of 2D maps and line graphs, is essential for a thorough and accurate representation of the simulation. This approach ensures a comprehensive understanding, validation, and interpretation of the acoustic field and its effects, which are critical for understanding the knowledge in HIFU therapy. We hope this detailed justification convinces the reviewer of the necessity of these figures in our publication.

Also, we would like to clarify that the figure regarding the pressure field (2D and 1D) is shown only once in the paper to provide complementary information about the pressure acoustic physics. We acknowledge that both the pressure field and intensity represent similar concepts. Therefore, in Figures 15 and 16, which present results for a realistic clinical approach, we did not include the results of pressure acoustics. However, as we have elaborated, we believe that demonstrating the pressure oscillation can be beneficial for readers to understand the physics of focused ultrasonics. We kindly request the reviewer to accept the inclusion of all figures in this format and not to delete any of them.

11. Incorporate line numbers of the revised manuscript to address all above comments within the rebuttal.

- **Response:** In addition to the track changes that highlight modifications in the manuscript, the line numbers of the modified areas are mentioned at the end of each response, as requested.
