# Supplementary material for: A comprehensive numerical procedure for high-intensity focused ultrasound ablation of breast tumour on an anatomically realistic breast phantom
Source: PLoS One. 2024 Oct 1;19(10):e0310899. doi: 10.1371/journal.pone.0310899 (PMC11444401; doi:10.1371/journal.pone.0310899)
Supplement: Attachment — Submitted filename: Reviewer 2 response.docx [file pone.0310899.s008.docx]

I appreciate the author’s effort in defending respective comments and accordingly addressed all comments satisfactorily. Additionally the following points need to clarify and revise for improvement.

1. In response to 1^st^ comment from authors: “Recognizing this limitation, our study introduces a novel all-in-one 3D FEM-based simulation approach using an anatomically realistic breast phantom”. The authors should carefully revise the term ‘novel work’ in the whole manuscript as the literature shows works on MRI based 3D modeling of anatomically realistic breast tissue.
2. I am not fully satisfied with the response to of 2^nd^ comment: ‘carefully site all possible references in support of explanation on how present numerical work will be helpful in solving any ***current clinically translational research study’***,
   a. one of the authors response is ‘This helps ***clinicians*** tailor HIFU parameters to each ***patient's anatomy***, resulting in more effective and personalized treatment plans [59] Li F, Feng R, Zhang Q, Bai J, Wang Z. Estimation of HIFU induced lesions in vitro: Numerical simulation and experiment. Ultrasonics. ***2006***;44:e337-e40’, please justify how the paper presents a current clinical challenge, also the referred paper is not a clinical study, it’s an in-silico and in-vitro study, and there is no patient anatomy related information.
   b. I appreciate referring clinical studies in response to 2^nd^ comment. In response the authors referred one review article and others are in-silico and in-vitro and ex-vivo studies.
3. Also the authors need to revise this line ‘Validation against experimental data and literature confirms our model's reliability and accuracy’ as it can create confusion to the reader that the ‘experimental work’ is performed in present manuscript.
