# Supplementary material for: A comprehensive numerical procedure for high-intensity focused ultrasound ablation of breast tumour on an anatomically realistic breast phantom
Source: PLoS One. 2024 Oct 1;19(10):e0310899. doi: 10.1371/journal.pone.0310899 (PMC11444401; doi:10.1371/journal.pone.0310899)
Supplement: Attachment — Submitted filename: Response to reviewer2.docx [file pone.0310899.s009.docx]

**Response to Reviewer 2 Comments**

**Manuscript ID: PONE-D-24-02187R2**

**Title:** A Comprehensive Numerical Procedure for High-Intensity Focused Ultrasound Ablation of Breast Tumour on an Anatomically Realistic Breast Phantom

**Authors:** Reza Rahpeima, Chao-An Lin

**Journal:** Plos One

We thank the reviewer for these constructive comments and have made the suggested modifications to the manuscript accordingly. Based on the reviewer’s comments, new materials are added to the paper. Below is an itemized list of all the implemented changes:

**Reviewer comments:**

1. In response to 1st comment from authors: “Recognizing this limitation, our study introduces a novel all-in-one 3D FEM-based simulation approach using an anatomically realistic breast phantom”. The authors should carefully revise the term ‘novel work’ in the whole manuscript as the literature shows works on MRI based 3D modeling of anatomically realistic breast tissue.

- **Response:** Thank you for the comment. As you requested all “novel” words are eliminated from the manuscript.

2. I am not fully satisfied with the response to of 2nd comment: ‘carefully site all possible references in support of explanation on how present numerical work will be helpful in solving any current clinically translational research study’,
a) one of the authors response is ‘This helps clinicians tailor HIFU parameters to each patient's anatomy, resulting in more effective and personalized treatment plans [59] Li F, Feng R, Zhang Q, Bai J, Wang Z. Estimation of HIFU induced lesions in vitro: Numerical simulation and experiment. Ultrasonics. 2006;44:e337-e40’, please justify how the paper presents a current clinical challenge, also the referred paper is not a clinical study, it’s an in-silico and in-vitro study, and there is no patient anatomy related information.

- **Response:** Thank you for your comment. The reference [59], Li F, Feng R, Zhang Q, Bai J, Wang Z. "Estimation of HIFU …," is not the correct citation for the manuscript. We believe you may have seen it incorrectly. The sentence “This helps clinicians tailor HIFU parameters to each patient's anatomy, resulting in more effective and personalized treatment plans” is actually cited by reference [64], De Maio A, Alfieri G, Mattone M, Ghanouni P, Napoli A. "High-Intensity Focused Ultrasound Surgery for Tumor Ablation: A Review of Current Applications." Radiology: Imaging Cancer. 2023;6(1). Anyway, regarding how our present numerical work helps address the clinical challenges highlighted in this review:

The review by De Maio et al. (2023) provides relevant insights into current clinical challenges. It emphasizes the importance of precise delivery and customization of HIFU settings based on the patient's specific anatomy and tumor characteristics. Factors such as tumor location, surrounding tissue properties, and the presence of barriers like fat, bone, or respiratory movement can affect HIFU efficacy. Clinicians need to account for these variables to optimize the treatment trajectory, energy delivery, and real-time monitoring to ensure complete ablation while minimizing damage to healthy tissues.

In our paper, this clinical challenge is addressed through our simulation platform, which helps predict the effects of HIFU based on patient-specific anatomical data. This allows clinicians to better tailor HIFU parameters, including lesion positioning, energy settings, and exposure durations, providing a more personalized approach to each patient’s treatment. This approach aligns with the challenges and recommendations identified in De Maio et al.’s review, emphasizing the need for patient-specific adjustments in HIFU therapy.

A brief summary of these explanations has been added to the manuscript as follows:

This paper emphasizes the importance of customizing HIFU settings based on the patient's anatomy and tumor characteristics. Factors like tumor location, tissue properties, and barriers such as fat or bone can affect treatment effectiveness, challenges that our simulation platform can address.

b. I appreciate referring clinical studies in response to 2nd comment. In response the authors referred one review article and others are in-silico and in-vitro and ex-vivo studies.

- **Response:** Thank you for your comment. The [64] and [65] references are review papers that include several clinical studies. We believed these would provide a more comprehensive overview; however, as you requested, we have added two specific clinical studies to the manuscript as follows:

Wu et al. conducted a randomized clinical trial on the use of HIFU for treating localized breast cancer, demonstrating its effectiveness in inducing tumor necrosis with minimal side effects [66]. Similarly, Peek et al. investigated the use of HIFU for treating fibroadenomata, showing significant reductions in lump size and pain, positioning it as a promising non-invasive alternative to surgery [67]. Our numerical platform for HIFU simulations can enhance these clinical findings by optimizing treatment parameters such as energy delivery and targeting accuracy, facilitating the development of efficient, patient-specific treatment strategies while addressing current clinical limitations.

3. Also, the authors need to revise this line ‘Validation against experimental data and literature confirms our model's reliability and accuracy’ as it can create confusion to the reader that the ‘experimental work’ is performed in present manuscript.

- **Response:** Thank you for your comment. We have modified the sentence accordingly to eliminate any potential confusion. The revised paragraph in the manuscript is as follows:

Validation using previously published experimental data and literature confirms the reliability and accuracy of our model, boosting clinical confidence in 3D simulations for HIFU treatment planning and outcome prediction, and encouraging the adoption of HIFU in clinical practice
